# Efficacy of different methods used for dry socket management: A systematic review

**DOI:** 10.4317/medoral.20589

**Published:** 2015-06-27

**Authors:** Maria Taberner-Vallverdú, Mariam Nazir, M. Ángeles Sánchez-Garcés, Cosme Gay-Escoda

**Affiliations:** 1 Dentistry student, School of Dentistry, University of Barcelona, Barcelona, Spain; 2 MD, DDS, MS, PhD, EBOS, Associated professor of Oral Surgery. Master’s Degree Program in Oral Surgery and Implantology, School of Dentistry, University of Barcelona. Researcher of the IDIBELL institute, Barcelona, Spain; 3 MD, DDS, MS, PhD, EBOS, Chairman and Professor of Oral and Maxillofacial Surgery, School of Dentistry, Barcelona. Director of the Master’s Degree Program in Oral Surgery and Implantology (EFHRE International University/FUCSO). Coordinator/Researcher of the IDIBELL Institute. Head of the Oral Surgery, Implantology and Maxillofacial Surgery Department of the Teknon Medical Center, Barcelona, Spain

## Abstract

**Background:**

Dry socket is one of the most common complications occurring after the extraction of a permanent tooth, but in spite of its high incidence there is not an established treatment for this condition. Objectives: Analyze the efficacy of different methods used in the management of dry socket regarding results of pain’s relief and alveolar mucosa healing compared to conventional surgical treatment of curettage and saline irrigation.

**Material and Methods:**

A Cochrane and PubMed-MEDLINE database search was conducted with the search terms “dry socket”, “post-extraction complications”, “alvogyl”, “alveolar osteitis” and “fibrynolitic alveolitis”, individually and next, using the Boolean operator “AND”. The inclusion criteria were: clinical studies including at least 10 patients, articles published from 2004 to 2014 written in English. The exclusion criteria were case reports and nonhuman studies.

**Results:**

11 publications were selected from a total of 627. Three of the 11 were excluded after reading the full text. The final review included 8 articles: 3 prospective studies, 2 retrospective studies and 3 clinical trials. They were stratified according to their level of scientific evidence using the SORT criteria (Strenght of Recommendation Taxonomy).

**Conclusions:**

All treatments included in the review have the aim to relief patient’s pain and promote alveolar mucosa healing in dry socket. Given the heterogeneity of interventions and the type of measurement scale, the results are difficult to compare. Curettage and irrigation should be carried out in dry socket, as well as another therapy such as LLLT, zinc oxide eugenol or plasma rich in growth factors, which are the ones that show better results in pain remission and alveolar mucosa healing. Assessment alveolar bone esposure must be a factor to consider in future research. Taking into account the scientific quality of the articles evaluated, a level B recommendation is given for therapeutic interventions proposed for the treatment of dry socket.

**Key words:**Dry socket, post-extraction complications, alvogyl, alveolar osteitis, fibrynolitic alveolitis.

## Introduction

Dry socket, is the most common complication following a dental extraction (1) and one of the most studied complications in dentistry (2). There are up to 17 different definitions for the clinical diagnosis of dry socket (3). Blum described dry socket as the presence of “postoperative pain in and around the extraction site, which increases in severity at any time between one and three days after the extraction, accompanied by a partially or totally disintegrated blood clot within the alveolar socket, with or without halitosis” (4) excluding any other cause of pain on the same side of the face (4).

Its incidence is approximately 3% for all routine extractions and can reach over 30% for impacted mandibular third molars (5), and many factors have been cited as contributing to the occurrence of dry socket including difficult or traumatic extractions, female sex, tobacco use, oral contraceptives and preexisting infection (6).

It has been suggested that an increased local fibrinolytic activity is the main etiological factor of dry socket. The increase in fibrinolytic activity could result in a premature loss of the intraalveolar blood clot after extraction (7). The fibrinolysis is the result of plasminogen pathway activation, which can be accomplished via direct (physiologic) or indirect (non physiologic) activator substances. Direct activators are released after trauma to the alveolar bone cells. Indirect activators are secreted by bacteria (8). Apart from the relation with the fibrinolytic process the exact etiology of dry socket is not well understood (9,10).

The treatment of alveolitis depends on each professional’s clinical experience (11) mainly due to the fact of its complex etiology, although many authors have published research on the management of dry socket.

The Cochrane Collaboration published a review on the local interventions for the management of dry socket, concluding there was no evidence to support any of the interventions included for its treatment (12).

The aim of this systematic review is to analyze the different methods used in the management of dry socket regarding results of pain’s relief and socket healing and the key question to meet the objective was: which medical treatment over conventional surgical treatment of curettage and irrigation with saline gets a faster remission of the intensity and duration of pain? and secondarily, which treatment promotes alveolar mucosa healing more effectively?.

## Material and Methods

A Cochrane and PubMed-MEDLINE databases search of articles was conducted between October 2013 and February 2014. The key words “dry socket”, “post-extraction complications”, “alvogyl”, “alveolar osteitis” and “fibrynolitic alveolitis” were used. Next, the terms were merged using the Boolean operator “AND”, in order to obtain the articles that included two or more of the used search terms.

The inclusion criteria were clinical studies including at least 10 patients published from 2004 to 2014 written in English. The exclusion criteria were case reports and non human studies.

The articles selection was agreed by consensus between two of the authors; first by reading of the titles and abstracts and, in those which seems relevant to identify whether they fulfilled the inclusion criteria or not.

## Results

**Figure 1:**
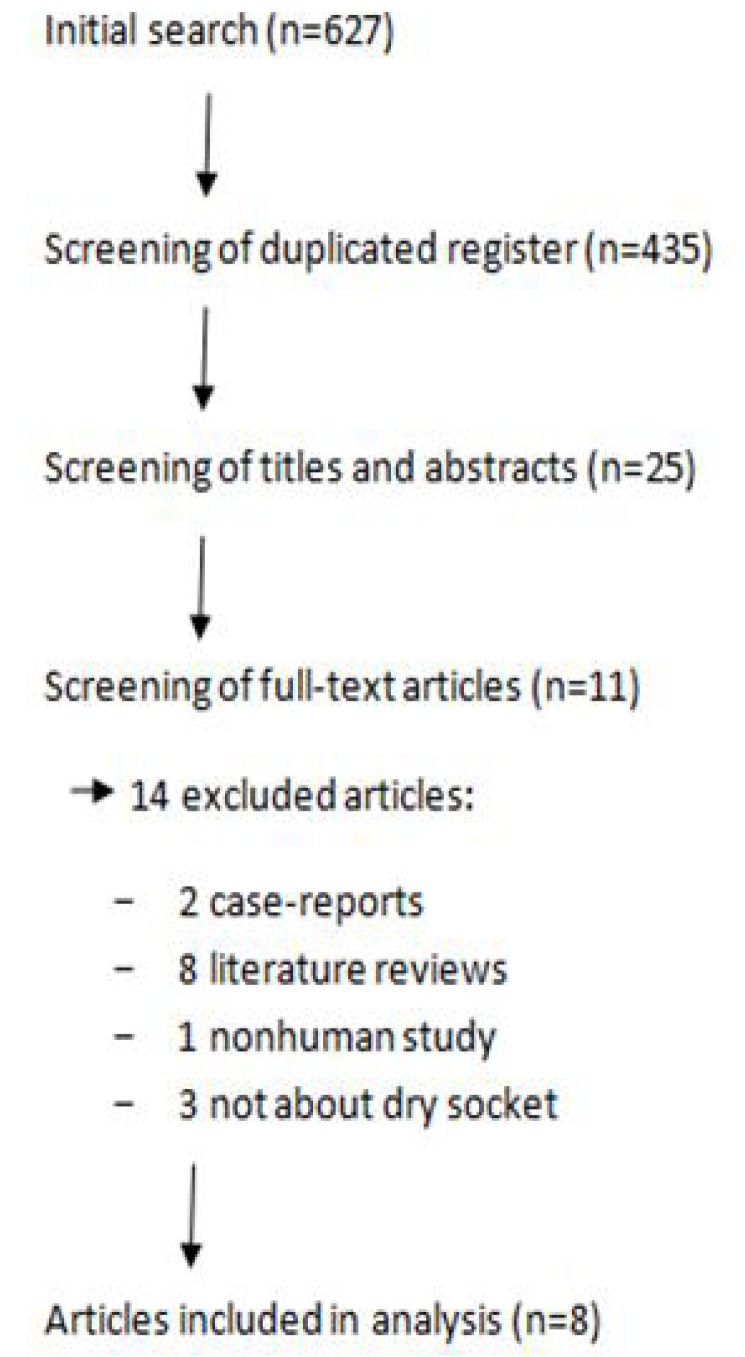
Flow of articles through the systematic review.

Out of the 627 studies obtained initially from the search, the complete text of 11 articles was analyzed. Three of these 11 articles were excluded due to the lack of direct relationship with the subject and finally, 8 articles with relevance were selected to be included in the systematic review: 3 prospective studies, 2 retrospective studies and 3 clinical trials (Fig. 1).

The articles were stratified according to their level of evidence, using the SORT criteria (Strenght of Recommendation Taxonomy) (13) ( Table 1 1, Table 2), resulting in 2 articles with a scientific evidence level of 1 and 6 with a scientific evidence level of 2.

**Table 1:**
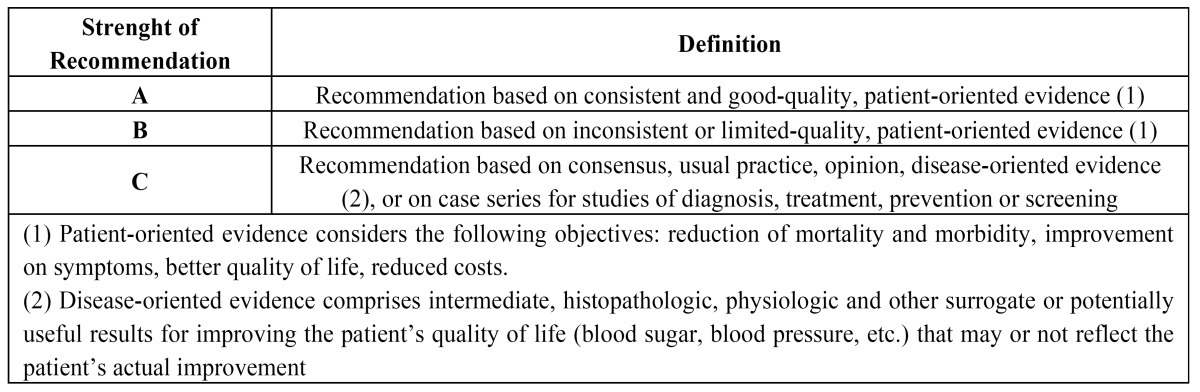
SORT Criteria (Strength of Recommendation Taxonomy) (13).

**Table 2:**
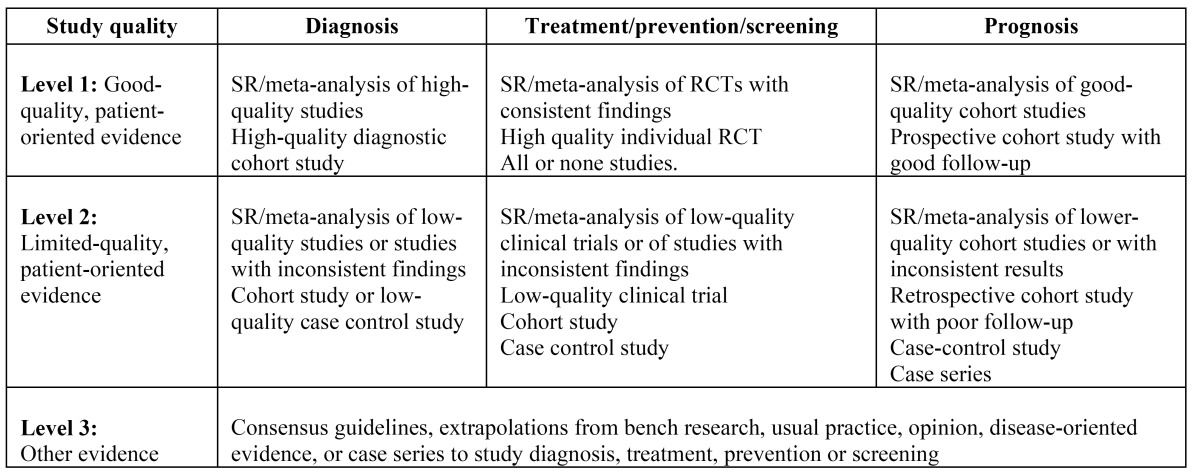
Levels of scientific evidence SORT (13).

The articles included in our review analyze the effectiveness of 8 different methods for the management of dry socket, represented in  Table 3.

**Table 3:**
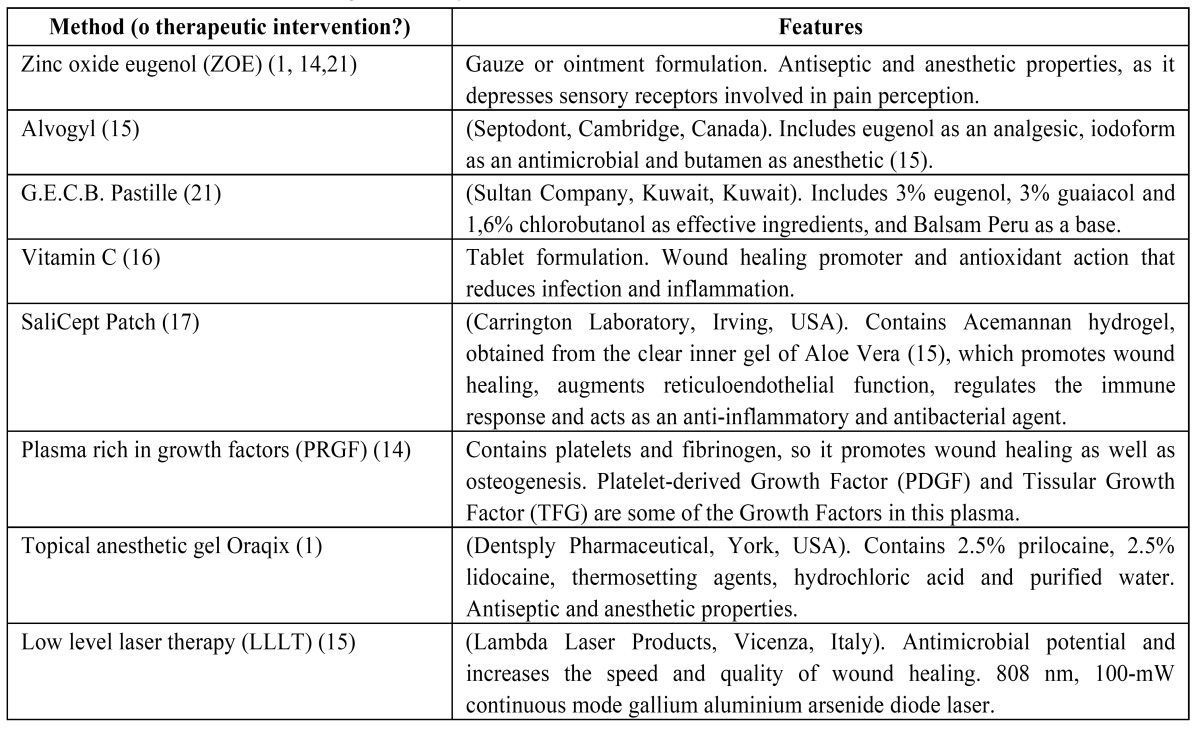
Different methods in the management of dry socket.

Curettage and irrigation are applied to almost all groups studied in the articles included in our review (1,14-16,19), as this seems to be imperative to remove debris, sequestra, and bacteria from the denuded bone (20) as a unic or as a control treatment or before appliying some local therapy.

The studies included in this revision compare the different treatments on two variables:

a) Pain’s relief.

As pain is the main symptom of this pathology, nine of the selected articles analyze the patient’s pain in dry socket and compare different treatments aimed to achieve pain remission (1,15,16,19,21-23).

To assess changes in pain’s intensity, some of the studies used the Visual Analogue Scale, asking the patients to measure their pain ranging from 0 (“no pain at all”) to 10 (“the most pain imaginable”), although some studies considered 8 or 9 the maximum level of pain (14). Other methods used to assess pain remission were the number of analgesic tablets needed (1), the mg of acetaminophen (21) or the percentage of patients who referred a pain decrease (16). All these results are quantitatively reflected on  Table 1.

a) Alveolar mucosa healing and alveolar bone exposition.

Alveolar mucosa healing is one of the most used signs to assess dry socket remission, what is more objectified than pain’s relief.

Only three of the studies (14,15,22) including quantitative references of the alveolar mucosa healing evolution in those alveoli that had developed dry socket. Socket healing was measured with different scales:

- One study observed granulation of the alveoli between 3 and 5 days of starting treatment in those patients who had taken 1 mg 8 hourly 24 hours post-extraction (19).

- Haraji *et al*. (22) use a gradation to assess alveolar post-extraction complications: 1: clot degeneration, 2: wound departure with pus, 3: wound departure without pus, 4: no healing.

- Pal *et al*. (14) punctuate alveolar healing as, 0: no healing, no clot formation, 0.5: clot formed/seen, 1: clot stabilized, 1.5: ½ of socket epithelialized and covered, 2: 2/3 of socket epithelialized and covered, 2.5: epithelialization almost complete, wound closed, 3: socket appears closed with normal mucosa coverage.

The results of these three studies according to their rating scale are shown in  Table 4 and  Table 4 continue.

**Table 4:**
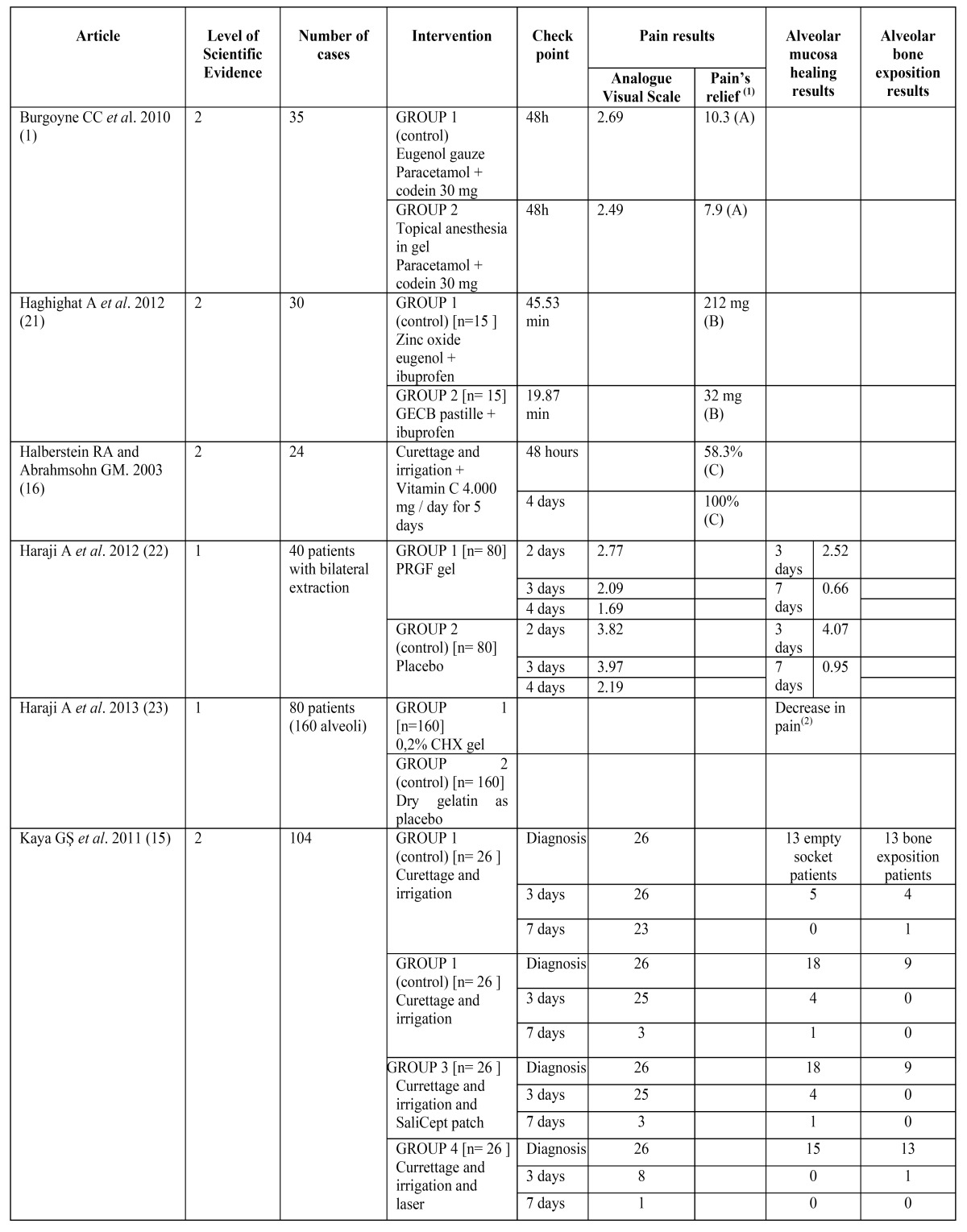
Results of the articles included in the systematic review (G.E.C.B.: Guaiacol, Eugenol, Chlorobutanol and Balsam Peru mixture. CHX: Chlorhexidine. P.R.G.F.: Plasma rich in Growth Factors. (1): (A): = number of analgesic tablets needed, (B) = ibuprofen mg needed, (C) =patients % with pain relief. (2): no quantitative reference.

**Table 4 Continue:**
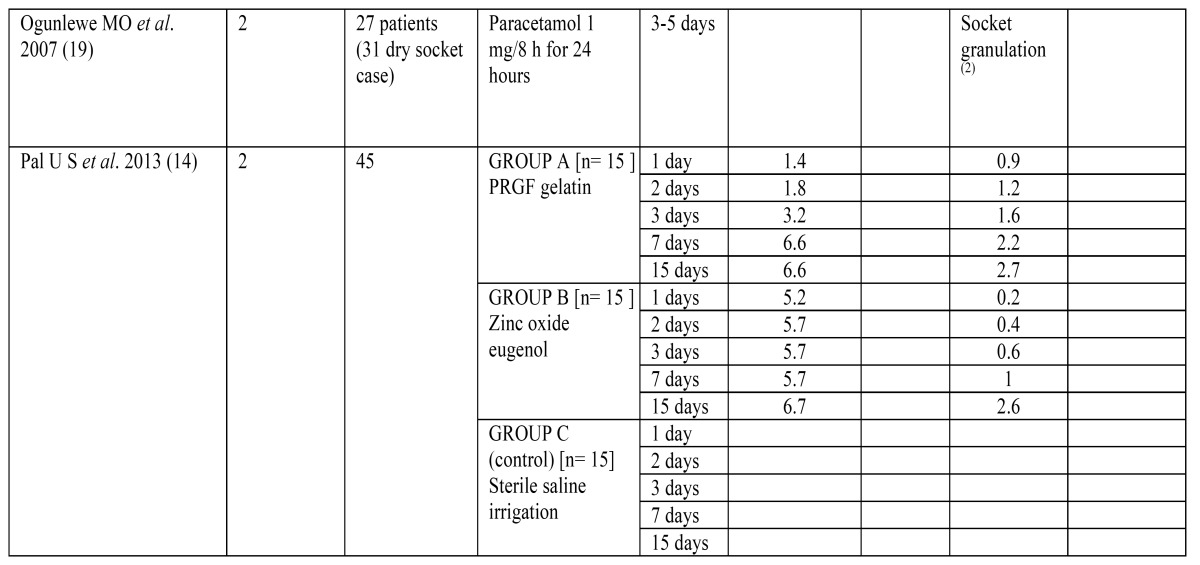
Results of the articles included in the systematic review (G.E.C.B.: Guaiacol, Eugenol, Chlorobutanol and Balsam Peru mixture. CHX: Chlorhexidine. P.R.G.F.: Plasma rich in Growth Factors. (1): (A): = number of analgesic tablets needed, (B) = ibuprofen mg needed, (C) =patients % with pain relief. (2): no quantitative reference.

Alveolar bone exposition (socket empty) is another useful parameter to determine dry socket healing, but it is a parameter only included in Kaya *et al*. study (15).

## Discussion

- Pain Remission

Given the disparity of interventions and the type of measurement scale, the results are difficult to compare between them.

According to 48 hours values from treatment initiation topical anesthetic gel is more effective than eugenol (1). Another method used in pain control is plasma rich in growth factors (PRGF) (22), producing a reduction in pain respect topical anesthesia in the first two days, but from the second day the difference between the two treatments decreases significantly (*P*<0.00 for each post-extraction day). As the highest pain intensity in dry socket appears between 48-72 hours post-extraction, then it can be assumed that PRGF is more effective in pain control, due because it produces a significant pain remission specially from the second day of extraction (22).

There is only one study treating pain with a guaiacol, eugenol, chlorobutanol and balsam peru (G.E.C.B.) pastille (21) or vitamin C (16). The first one concludes that although traditional treatment with zinc oxide eugenol is acceptable, G.E.C.B. pastille has a faster effect (pain remission in 19.87 minutes after G.E.C.B. pastille instead of the 45.53 minutes after zinc oxide eugenol), and the second article concludes that a 4000 mg vitamin C dose along with curettage and irrigation achieves a 100% pain remission in just 4 days, although it has to be pointed out that there was no control group to which the results were compared.

Kaya *et al*. (15) conducted a randomized clinical trial with the aim to compare the effectiveness of Alvogyl, SaliCept and low-level laser therapy (LLLT) in pain reduction in dry socket, and concluded that LLLT performed superiorly to SaliCept and alvogyl and achieved a pain remission in the third day. The intensity of pain decreased more rapidly in all three treatment groups than in the control group (*P*<0.05), treated with curettage and irrigation alone.

Ogunlewe *et al*. (19) recommend pharmacological treatment in combination with curettage and saline irrigation. In their prospective study a 1 mg acetaminophen dose was prescribed 2 hours post operatively, and then 1 mg 8 hourly for the next 24 hours. Satisfactory results were obtained with this regimen, but the article does not show any quantitative references, and the treatment was not compared with any other method.

- Alveolar mucosa healing 

Results obtained in the studies are hardly comparable because of the different measurement scale, but the time when alveolar mucosa healing was complete can be assessed in three studies (14,15,22).

According to two of the studies, PRGF in gel formulation produces a faster and better alveolar mucosa healing, being almost complete 15 days of starting treatment (14,22), a little bit earlier than in zinc oxide eugenol group (*P*<0.01).

Regarding the different therapies compared in the study of Kaya *et al*. (15), none of the patients treated with low-level laser therapy had empty socket after three days of application, so all had begun the healing process, while in the group treated with Alvogyl little more than half of the patients had started the process (even less than in the control group, which was only performed curettage and irrigation).

- Alveolar bone exposure

In Kaya *et al*. study alveolar bone exposure decreased more rapidly in the SaliCept patch treated group, as in three days none of the patients had alveolar bone exposure. Results of LLLT group were also significant (92% patients didn’t have alveolar bone exposition in the 3rd day). Curiously, alveolar bone exposure decrease was higher in the control group (69,2%) than in Alvogyl treated group (40%) (*P* > 0.05, standard error 0.117) (15).

We can mention finally that there are other publications that propose some measures to prevent dry socket, including the use of topical antibiotics after third molars extraction (24) or oral antibiotics before dental extraction (25). Various items support the preventive effect on dry socket of chlorhexidine. In the meta-analysis by Caso *et al*. (26) they conclude that rinsing with chlorhexidine on the day of the extraction and in subsequent days may reduce the incidence of dry socket, a result corroborated in the study of Hedström and Sjögren (27). More recently, Hita-Iglesias *et al*. (28) have shown a greater efficacy of chlorhexidine in gel formulation versus rinse on dry socket’s prevention, because of the longer chlorhexidine bioavailability in the applied area with gel formulation.

In regards to the key questions of the article, only two articles compare a medical treatment over curettage and irrigation alone as a control (14,15). In both articles comparison was made through visual analogue scale. Kaya *et al*. (15) performed alveolar curettage followed by through irrigation with a sterile saline solution (0.09% NaCl). All debris was removed, taking care to avoid dislodging any normal clot found in the socket. Curettage and saline irrigation were repeated again three days later. The intensity of pain decreased more rapidly in all three treatment groups than in the control group (*P*<0.5). This decrease was significantly greater for the LLLT group than for the Alvogyl, SaliCept patch and control group. Pal *et al*. (14) permormed only irrigation with saline for the control group of their study without any other manipulation of the socket or any farmac and in terms of pain relief, zinc oxide eugenol dressing was more effective than others treatments tested and the control group.

In terms of which treatment promotes alveolar mucosa healing more effectively, it can be concluded that LLLT theraphy and PRGF obtain the fastest healing, according to the results of the three articles that assess it (14,15,22).

## Conclusions

All treatments included in the review aim to relief patient’s pain and promote alveolar mucosa healing in dry socket. Given the disparity of interventions and the type of measurement scale, the results are difficult to compare between them. Curettage and irrigation of the socket should be carried out in dry socket, as well as another therapy such as LLLT, zinc oxide eugenol or plasma rich in growth factors, which are the ones that show better results in pain remission and alveolar mucosa healing.

Assessment of bone exposure must be a factor to consider in future research and could be complementary to alveolar mucosa healing and therefore give a more complete view of the efficacy of the different methods used for dry socket treatment.

After the article’s analysis and according to their scientific quality, a level B recommendation is given to all of the therapeutic interventions proposed for dry socket’s treatment.
